# Quantitative cross-validation and content analysis of the 450k DNA methylation array from Illumina, Inc.

**DOI:** 10.1186/1756-0500-5-210

**Published:** 2012-04-30

**Authors:** Jessica Roessler, Ole Ammerpohl, Jana Gutwein, Britta Hasemeier, Sumadi Lukman Anwar, Hans Kreipe, Ulrich Lehmann

**Affiliations:** 1 Institute of Pathology, Medizinische Hochschule Hannover, Carl-Neuberg-Str. 1, D-30625, Hannover, Germany; 2 Institute of Human Genetics, University Hospital Schleswig Holstein, Kiel, Germany

## Abstract

**Background:**

The newly released 450k DNA methylation array from Illumina, Inc. offers the possibility to analyze more than 480,000 individual CpG sites in a user friendly standardized format. In this study the relationship between the β-values provided by the Illumina, Inc. array for each individual CpG dinucleotide and the quantitative methylation levels obtained by pyrosequencing were analyzed. In addition, the representation of microRNA genes and imprinted loci on the Illumina, Inc. array was assessed in detail. Genomic DNA from 4 human breast cancer cell lines (IPH-926, HCC1937, MDA-MB-134, PMC42) and 18 human breast cancer specimens as well as 4 normal mammary epithelial fractions was analyzed on 450k DNA methylation arrays. The β-values for 692 individual CpG sites from 62 different genes were cross-validated using conventional quantitative pyrosequencing.

**Findings:**

The newly released 450k methylation array from Illumina, Inc. shows a high concordance with quantitative pyrosequencing if identical CpG sites are analyzed in cell lines (Spearman r = 0.88, p ≪ 0.0001), which is somewhat reduced in primary tumor specimens (Spearman r = 0.86, p ≪ 0.0001). 80.7% of the CpG sites show an absolute difference in methylation level of less than 15 percentage points. If different CpG sites in the same CpG islands are targeted the concordance is lower (r = 0.83 in cell lines and r = 0.7 in primary tumors). The number of CpG sites representing microRNA genes and imprinted loci is very heterogeneous (range: 1 – 70 CpG sites for microRNAs and 1 – 288 for imprinted loci).

**Conclusions:**

The newly released 450k methylation array from Illumina, Inc. provides a genome-wide quantitative representation of DNA methylation aberrations in a convenient format. Overall, the congruence with pyrosequencing data is very good. However, for individual loci one should be careful to translate the β-values directly into percent methylation levels.

## Background

The genome-wide assessment of DNA methylation patterns becomes more and more important in cancer research [1,2]. However, so far the comprehensive analysis of all potential CpG sites in the human genome, as demonstrated for the first time in 2009 by Lister et al. [3] is - in terms of costs per sample and required data processing resources - well beyond the options of most research groups. Therefore, various protocols have been developed to analyze a representative subset of the human genome [4,5]. The most recent addition to this arsenal of methods is the 450k DNA methylation array from Ilumina. Inc, which measures in parallel the methylation of approximately 480,000 CpG sites across the human genome. It was attempted to cover all known protein coding genes as well as a fairly large number of non-coding RNA genes and imprinted loci. Based on the well-established and widely used Infinium technology [6] this array platform promises an easy-to-use, standardized, and cost effective format for the analysis of a representative subset of the human methylome.

The array design, content, and technical performance are well described in two recent publications [7,8]. However, both publications are not independent from the manufacturer and so far only limited data are available about cross-validation of the 450k methylation array with other quantitative methods well established in the field of DNA methylation research. Dedeurwaerder et al. [9] compared the methylation level of altogether 15 CpG sites in 2 cell line samples (30 measurements) and 4 CpG sites in 6 primary samples (6 measurements) with quantitative pyrosequencing. An analysis of the representation of genes important in tumor biology (measured as number of CpG sites per gene) on the 450k array has not yet been published so far. Therefore, we compared the methylation level of altogether 692 individual CpG sites from 62 different genes in a series of cell lines and primary human tissue samples and analyzed in detail how microRNA genes and imprinted loci are represented on the array.

## Findings

It is expected that the 450k DNA methylation array from Illumina, Inc. will be as widely used as its predecessor, the 27k methylation array, and will thereby influence the methylation field to a great extent. Therefore, careful and critical analysis of the strengths and weaknesses of this methodology as early as possible is of great importance.

### Cross-validation of 450k methylation array and pyrosequencing

For the cross-validation of the 450k array we selected quantitative pyrosequencing which is a well-established and widely used method in the field of DNA methylation research. Several groups, including our own [10,11], could show that for many loci pyrosequencing provides a very good quantitative measure of the methylation level at individual CpG sites (see also Additional file 1). The comparison of β-values and pyrosequencing results was performed separately for cell lines and primary human tissue samples and included altogether 692 individual CpG dinucleotides (340 in cell lines and 352 in primary specimens). From the 352 individual CpG sites compared one-by-one in primary tumor specimens 80.3% show a difference in methylation level of less than 15 percentage points. If 10 percentage points are chosen as threshold, 60.5% of all data points are within this range of agreement. The corresponding numbers for the analyses in cell lines are very similar: 77.4% and 63.8%, respectively.

**Figure 1:**
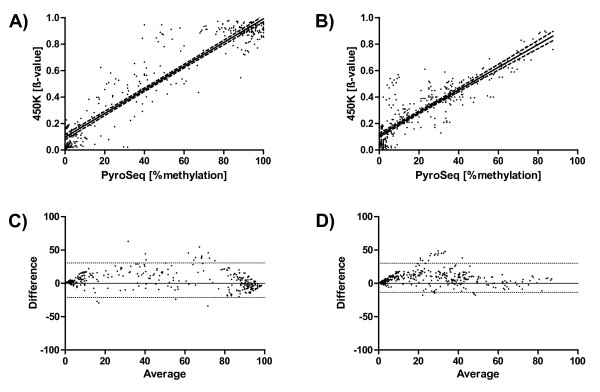
**Cross-validation of 450k methylation array and quantitative pyrosequencing.** Shown are scatter plots for the methylation values obtained by pyrosequencing (x-axis) and the β-values obtained from the 450k array for identical CpG sites. A) cell lines (Spearman r = 0.88, linear regression r^2^ = 0.89, slope: 0.89, 95% confidence interval: 0.86 – 0.93), B) primary human tumor tissues (Spearman r = 0.85, linear regression r^2^ = 0.77, slope: 0.86, 95% confidence interval: 0.81 – 0.91). Each dot in the figure represents the comparison of % methylation according to pyrosequencing versus the β-value for a single CpG site in one sample. C) and D) corresponding Bland-Altman plots. The difference between both methods for every individual measurement is plotted against the mean of both methods. The mean of the differences +/− two times the standard deviation denotes the 95% range for the limits of agreement (marked by the horizontal dotted lines). For the construction of these plots the β-values have been multiplied by the factor 100 in order to obtained data sets of the same size range. The coordinates of the individual CpG sites analyzed in this comparison are compiled in Additional file 3

Figure 1A and B) show a very good concordance between both methods (cell lines: Spearman r = 0.88, primary tissue Spearman r = 0.86). However, the calculation of a correlation coefficient or a regression coefficient can be misleading, because the scatter plots are dominated by two agglomerations of data points near the origin of the scatter plot and near the 100% value. In between, the accordance between both methods is reduced. This can be seen more clearly in the corresponding Bland-Altman-Plots (Figure 1C and D). In a Bland-Altman-Plot the difference between the two methods under study is plotted against the mean of both methods for every individual pair of measurements. The interval of the mean of the difference +/− two times the standard deviation defines the 95% interval of the limits of agreement. In this kind of plot it is much more obvious that the data points become more dispersed in the range of 25 – 75% methylation (indicating less concordance). All data points outside the 95% limits of agreement (marked by the dotted lines) are lying in this middle region. If the CpG sites targeted by the pyrosequencing assay and the 450k array are not identical but from the same CpG islands, the agreement is - as expected – reduced (Spearman r = 0.83 for cell lines and r = 0.71 for primary specimens, see Additional file 2).

Common SNPs could be excluded as a major source of the discrepancies between pyrosequencing and 450k methylation array. All data points above or below the limits of agreement in Figure 1C and D) were checked for the presence of SNPs in the corresponding probes on the 450k array and also in the pyrosequencing assays. As a control 20 data points with very high concordance were randomly selected and checked for the potential presence of SNPs. No clear correlation between the level of agreement and the potential presence of SNPs could be identified.

If a threshold of 10 to 20% methylation is chosen for scoring CpG sites as “hypermethylated”, this reduced accordance in the middle range of methylation levels (25 – 75%) is not a problem at all. However, if cluster analyses are performed with the β-values for subsets of probes, which show intermediate methylation levels, results should be interpreted carefully.

If only cell line DNA samples are used for the evaluation and comparison of methods for the analysis of DNA methylation a certain bias is introduced because the vast majority of loci display a clear dichotomous distribution in cell lines: either no or very low level DNA methylation or very high, nearly complete methylation. Therefore, any scatter plot (and subsequent calculations of correlation or regression coefficients) illustrating the comparison of methods or samples is dominated by two populations of data points (i.e., close to 0 and close to 1 or 100%). This can easily be verified by visual inspection of Fig. 6 A) and B) in Sandoval et al. [8], Fig. 4 in Bibikova et al., [7], Fig. 4 A) in Dedeurwaerder et al. [9], and our own Figure 1.

The first publication about the 450k array from Sandoval et al. [8] in collaboration with Illumina, Inc. gives a very good overview of the loci covered and the classes of CpG sites included in the 450k array design. However, it did not cross-validate the results directly with another method but only indirectly by comparing the 450k array with the well-established GoldenGate® array and the 27k array from Illumina, Inc. In the second publication about the 450k array exclusively from Illumina, Inc. [7] a cross-validation with deep-sequencing for two samples is reported. Due to the high number of data points (189,000 and 167,000, respectively) the correlation seems to be extremely good (r^2^ = 0.96). But a more careful inspection of Figure 5B from this publication shows that the data points form a “broad band”, which is 30 – 40 percentage points wide, meaning that many measurements differ by 30 – 40 percentage points. There are an unidentifiable number of measurements with no methylation in the sequencing analysis and up to 50% methylation level according to the array analysis. This is indicated by the black line at the bottom of Figure 5 B) parallel to the x-axis at 0.0 methylation value according to sequencing (y-axis) and ranging from a β-value of 0.0 to a β-value of approx. 0.5 at the x-axis. The same phenomenon can be seen at the upper limit of the scale at the top of Figure 5 B): A black line parallel to the x-axis at 1.0 methylation according to sequencing and ranging from a β-value of approx. 0.4 to a β-value of 1.0 indicating 100% methylation according to sequencing and 40 – 100% according to array analysis. This clearly shows in line with our results that whereas overall the concordance is very high the derivation of methylation levels of individual genes or loci from β-values might be uncertain and requires independent validation.

### Comparison of Infinium I and Infinium II assays

A potential problem of the 450k methylation array is the fact that it combines two different assays on a single array, namely the Infinium I and Infinium II technology (see Figure 1 in ref. [7] or Figure 2 in ref. [9] for a very good illustration of the principal of the two different assays). Dedeurwaerder et al. [9] describe in detail the effect of these two different assays on the β-value distribution (Fig. 1 in ref. [9]) observed now by many users in the DNA methylation community. Therefore, we analyzed all correlations between β-values and pyrosequencing results separately for Infinium I and II assays. Overall, the agreement between pyrosequencing and Infinium I and II, respectively, are very similar for both cell lines and primary patient samples (see Additional file 4). The above mentioned observation, that the concordance of both methods in the range of 25 – 75% methylation is reduced, affects both assay types to a similar extent. Our re-analysis of the data presented by Dedeurwaerder et al. [9] shows that the peak-correction proposed by these authors does not improve the congruence (see Additional file 5). These authors performed also a much more limited comparison of pyrosequencing and the 450k methylation array, especially for primary human tissue samples: The results of only six measurements are presented (Fig 5 B in Dedeurwaerder et al. [9]), in comparison to 352 measurements in primary tissues samples evaluated by us.

The reduced concordance in primary patient samples might be due to the heterogeneous methylation patterns frequently encountered in primary samples compared with cell lines. As described in detail by Bibikova et al. [7] in the introduction section of their publication the basic assumption for the Infinium assay design is, that adjacent CpG sites display very similar methylation levels, thereby enabling the selection of closely spaced probes including potential methylation sites in their binding site. However, the two studies cited by Bibikova et al. as supporting this assumption [12,13] analyzed healthy normal tissue samples or healthy primary cell samples but not tumor tissue samples. Since many genes and loci show extensive heterogeneity in methylation patterns in tumor cells (e.g., *p16*^*INK4A*^ in HCC [11]), the methylation level of individual CpG sites might be assessed incorrectly.

### Representation of microRNA genes and imprinted loci

Since we have a long standing interest in epigenetic regulation of microRNA genes [14] and imprinted loci [15] the representation of these two important classes of non-protein coding genes with pleiotropic regulatory functions was assessed in detail for several microRNA genes already under investigation in our group [11,14]. The number of CpG sites analyzed is in general comparable to the number of CpG sites included in pyrosequencing assays (i.e., 4 – 13 CpG sites). However, due to the assay design the CpG sites covered are spread over a much larger region: 900-1500 bp, in comparison to 50 – 100 bp for an average pyrosequencing assay. Whether this is an advantage or disadvantage depends on the circumstances. The often very heterogeneous methylation patterns encountered especially in primary tumor specimens might be better recognized by the analysis of a continuous stretch of CpG sites as with pyrosequencing or conventional bisulfite sequencing. On the other hand, the spreading of CpG sites analyzed on the 450k array might give a better representation of the methylation status of a whole genomic locus.

A few microRNA loci are very well represented on the 450k array (e.g., *hsa-mir-1256* with 34 CpG sites and *hsa-miR-548H4* with 70 CpG sites) despite the fact that so far only very limited data about epigenetic regulation or the cellular function of these microRNAs are available (status: January 31^th^, 2012). Notwithstanding sparse published functional evidences the *miR-548* family is represented by altogether 252 CpG sites. However, this might be advantageous for future studies focusing on these microRNAs.

**Figure 2:**
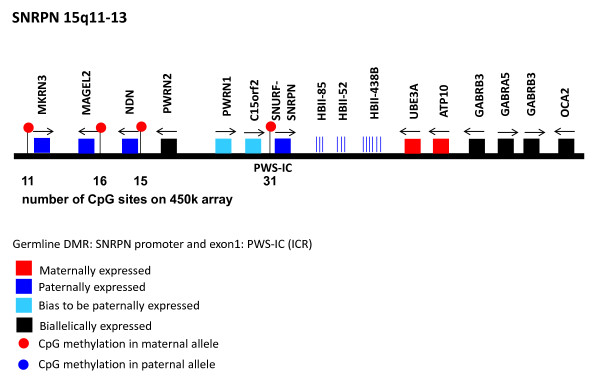
**Representation of the*SNRNP*locus on the 450k methylation array.** This figure shows the organization of the *SNRPN* locus with the four differentially methylated regions (red lollipops). Below each region the number of CpG sites represented on the 450k array is indicated (based on Robertson 2005 [16] and references therein)

The representation of imprinted loci is on average much better. Up to 180 CpG sites cover a single differentially methylated region. The analysis of the following 11 loci revealed a very good representation of these imprinted loci (in terms of number and location of CpG sites assayed on the 450k array): *IGF2, IGF2R, SNRPN, CDKN1C, MEG3/DLK1, GNAS, PEG3, PLAGL1(ZAC), PEG10, MEST(PEG1), GRB10*. Figure 2 shows as an example how many CpG sites within the four imprint control regions of the *SNRPN* locus on chr. 15q11-13 are measured using the 450k array.

**Figure 3:**
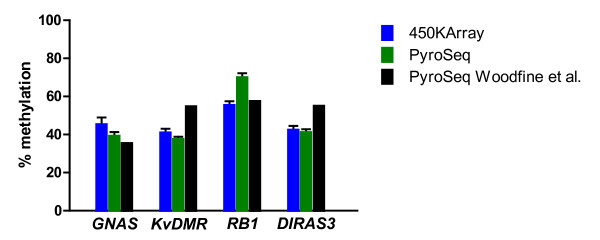
**Comparison of methylation levels for four imprinted loci.** The methylation level of *GNAS, KvDMR, RB1*, and *DIRAS3* was measured in four healthy normal mammary tissue samples using pyrosequencing and the 450k methylation array. Shown are the mean values, the error bars indicating the standard deviation. For *KvDMR* six CpG sites represented on the 450k array are measured by the pyrosequencing assay. For the remaining three loci only one CpG site was also measured by the pyrosequencing assay. In addition the data from Supplementary Table S2 from Woodfine et al. [17] are incorporated. These data are also obtained by pyrosequencing from 3 or 6 normal mammary tissue specimens. No standard deviations are reported for these data

The concordance between the β-values, our pyrosequencing results, and the pyrosequencing values reported by Woodfine et al. [17] for four imprinted loci is shown in Figure 3. This graph represents altogether 36 measurements (9 different CpG sites in four samples).

Despite the above discussed reduced agreement between both methods in the middle range of methylation levels for some loci the results in Figure 3 demonstrate a very good concordance for four imprinted loci in normal primary mammary tissue samples between the pyrosequencing data from two independent laboratories and 450k DNA methylation array data.

A potential pitfall of the analysis of 450k array data is the annotation of probes which is not really consistent and straightforward in the small set of microRNA loci which we investigated in detail. A purely gene name-based search will miss many microRNA gene associated CpG sites due to inconsistent nomenclature. The assignment of a given CpG dinucleotide to a microRNA is still very difficult or impossible due to the lack of knowledge about microRNA gene promoters and the primary microRNA transcripts and due to the fact that many microRNAs are located within protein coding genes. Nevertheless, several inconsistencies in nomenclature could be eliminated in the next version of the probe identification sheet.

It should also be mentioned that a major limitation of all bisulfite treatment based methodologies is the inability to differentiate between methylcytosine and hydroxymethylcytosine [18-20]. The latter one is playing a crucial role at least in developmental biology.

## Conclusion

The newly released 450k methylation array from Illumina, Inc. provides a genome-wide quantitative representation of DNA methylation aberrations in a convenient format. For the majority of CpG sites the β-values represent a very good measure of the methylation status. However, for individual loci the direct transformation of β-values into methylation levels should be handled with care and validated by an independent method. The representation and annotation of functionally important loci could be improved in future versions of the array.

## Methods

### Tissue specimens and bisulfite modification of DNA

All primary human tissue samples were retrieved from the archive of the Institute of Pathology, Hanover Medical School (Germany) and analyzed anonymously following the guidelines of the local Ethics committee ("Ethik-Kommission der Medizinischen Hochschule Hannover", head: Prof. Dr. Tröger). Tumor cell content was determined to be greater than 70%. DNA was isolated by digestion with Proteinase K (Merck, Darmstadt, Germany) followed by phenol/chloroform extraction from a total of 22 specimens ( Additional file 6). Genomic DNA (1 μg) from tumor specimens was treated with sodium bisulfite using the EZ DNA Methylation™ kit (ZymoResearch, Irvine, CA, USA) following the protocol supplied by the manufacturer with the exception of eluting the treated DNA with distilled water instead of using the provided elution buffer.

Cell lines HCC1937, MDA-MB-134, and PMC42 were purchased from ATCC and cultivated following the provided protocols. The cell line IPH-926 was established in our institution and is described comprehensively elsewhere [21].

### Methylation analysis using the 450k array

DNA methylation analysis using the Infinium HumanMethylation450k BeadChip (Illumina, Inc., San Diego, CA, USA) was performed according the manufactures' instruction. The HumanMethylation450 BeadChip was developed to assay more than 480,000 CpG sites selected CpG loci in parallel (Bibikova et al., 2011). DNA methylation data were processed using GenomeStudio software (ver. 2011.1; Illumina, Inc.) applying the default settings.

### Methylation analysis using pyrosequencing

PCR products were generated in a 25 μL reaction volume with 400 nmol/L of forward, 40 nmol/L reverse and 400 nmol/L universal biotinylated primers, 200 μmol/L of each dNTP, 1.5 mmol/L or 2.5 mmol/L MgCl_2_ (see Additional file 7 for all primer sequences and reaction conditions), 1x Platinum-Taq reaction buffer and 1.25 units PlatinumTaq™ (Invitrogen, Karlsruhe, Germany). PCR conditions were 95°C for 5 minutes, followed by 45 cycles with denaturation at 95°C for 30 seconds, annealing at 55°C or 60°C for 45 seconds, and elongation at 72°C for 30 seconds finished with 1 cycle final elongation at 72°C for 5 minutes. The reverse primer is tagged by a sequence recognized by the universal primer. Therefore, a single (expansive) biotinylated primer can be used for all different gene-specific assays [22].

PCR products (5–20 μL) were added to a mix consisting of 3 μL Streptavidin Sepharose HP™ (Amersham Biosciences, Freiburg, Germany) and 47 μL binding buffer (Qiagen, Hilden, Germany) and mixed at 1200 rpm for 5 minutes at room temperature.

Using the Vacuum Prep Tool™ (Qiagen, Hilden, Germany), single-stranded PCR products were prepared following the manufacturer's instructions. The sepharose beads with the single stranded templates attached were released into a PSQ 96 Plate Low™ (Qiagen, Hilden, Germany) containing a mix of 12 μL annealing buffer (Qiagen, Hilden, Germany) and 500 nmmol/L of the corresponding sequencing primer (see Additional file 7). Pyrosequencing™ reactions were performed in a PyroMark MD System (Qiagen, Hilden, Germany) according to the manufacturer's instructions using the PyroGold SQA™ Reagent Kit (Qiagen, Hilden, Germany). CpG site quantification was performed using the methylation Software Pyro Q-CpG™.

### Statistical analyses

All calculations were performed using GraphPad Prisms5 software. p-values smaller than 0.5 were considered statistically significant.

For the comparison of the two methods Bland-Altman-Plots were generated [23]. In these plots the difference of two methods is plotted against the average of both methods. A comprehensive description of this type of data presentation by Altman and Bland ("Measurements in Medicine: the Analysis of Method Comparison Studies", The Statistician 32 (1983) 307 – 317) can be found freely available at: https://person.hst.aau.dk/slc/Teaching/Papers/BlandAltman83.pdf(availabilitychecked: 17th February 2012). In order to obtain data sets of comparable range for the construction of the Bland-Altman-plots, the β-values were transformed using the following equation: β-value x 100 = % methylation (Illumina).

## Abbreviations

bp, Base pair.

## Competing interest

The authors declare that they have no competing interests.

## Authors’ contributions

UL, OA and JR conceived the study; JR and BH prepared all samples and performed all pyroseqeuencing measurements; JG and OA performed the 450k array hybridization; JR, JG, OA, SLA and UL analyzed the 450k and the pyrosequencing data; HK selected and evaluated all cases; UL and JR wrote the manuscript with support from HK, SLA, OA, and JG. All authors read and approved the final manuscript.

## Supplementary Material

Additional file 1 Linearity of pyrosequencing for 4 different genes (*SFRP1, APC, DAPK, KvDMR*) Click here for file

Additional file 2 Comparison of the concordance between pyrosequencing and the 450k array if the same CpG island is analyzed. For this purpose the mean methylation levels obtained by each method for a given CpG island were compared. Indicated are correlation coefficients according to Spearman (r) and linear regression coefficients (r^2^) Click here for file

Additional file 3 Coordinates for all individual CpG sites compared in Figure 1 Click here for file

4 Comparison of the concordance between pyrosequencing and the 450k array for Infinium I and II assays separately. Indicated are correlation coefficients according to Spearman (r) and linear regression coefficients (r^2^) Click here for file

Additional file 5 Scatter plot (A) and Bland-Altman-Plots without (B) and with (C) peak correction for the data from Dedeurwaerder et al. Table S2 Click here for file

Additional file 6 Primary human specimens used in this study Click here for file

Additional file 7 Sequence of all primers used in this study Click here for file
