# Supplementary figures and images for: Quantitative cross-validation and content analysis of the 450k DNA methylation array from Illumina, Inc
Source: BMC Res Notes. 2012 Apr 30;5:210. doi: 10.1186/1756-0500-5-210 (PMC3420245; doi:10.1186/1756-0500-5-210)

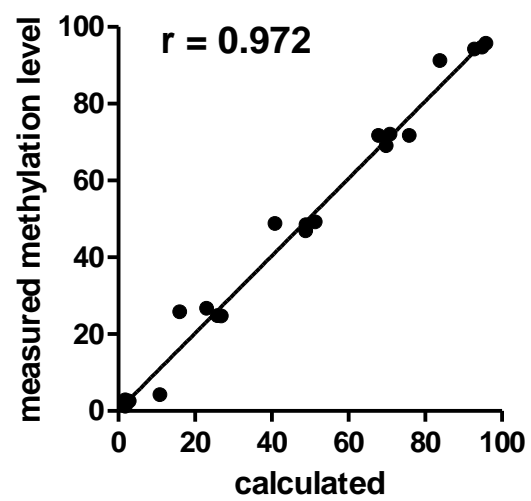

Supplement: Additional file 1 — Linearity of pyrosequencing for 4 different genes (SFRP1, APC, DAPK, KvDMR) [file 1756-0500-5-210-S1.pdf]

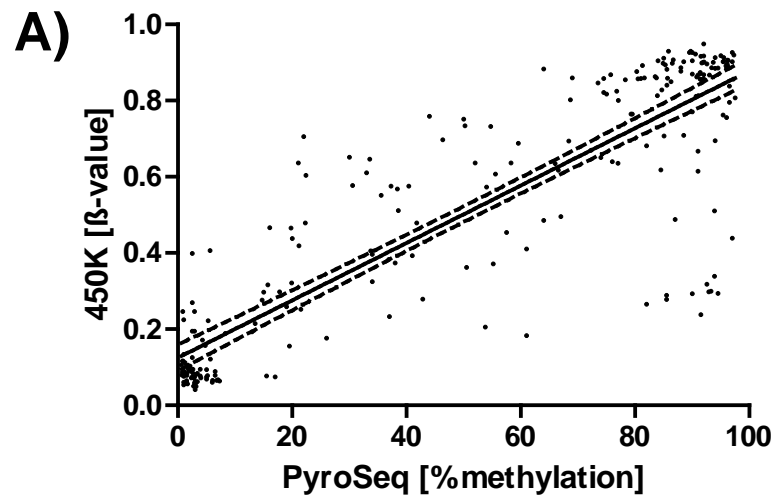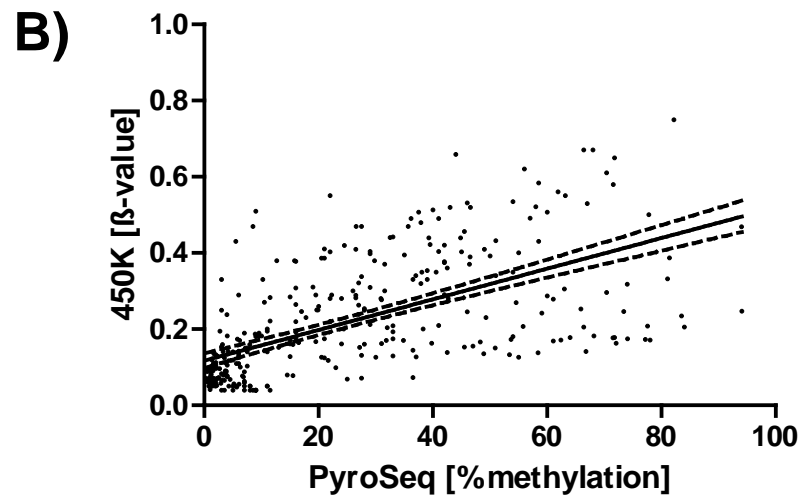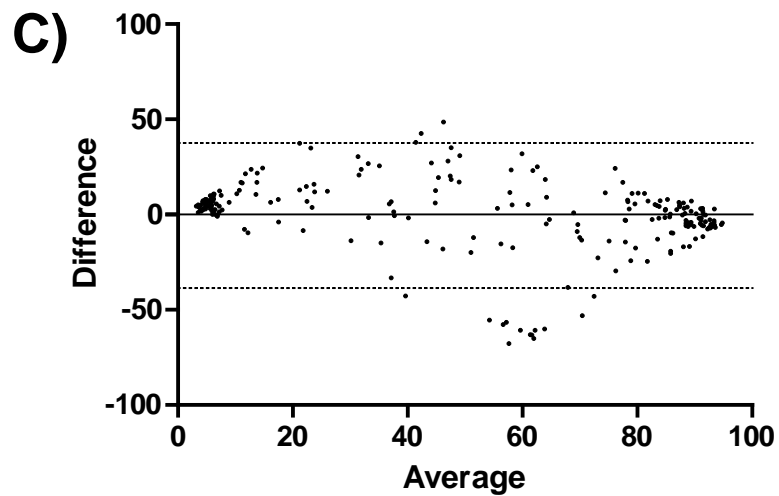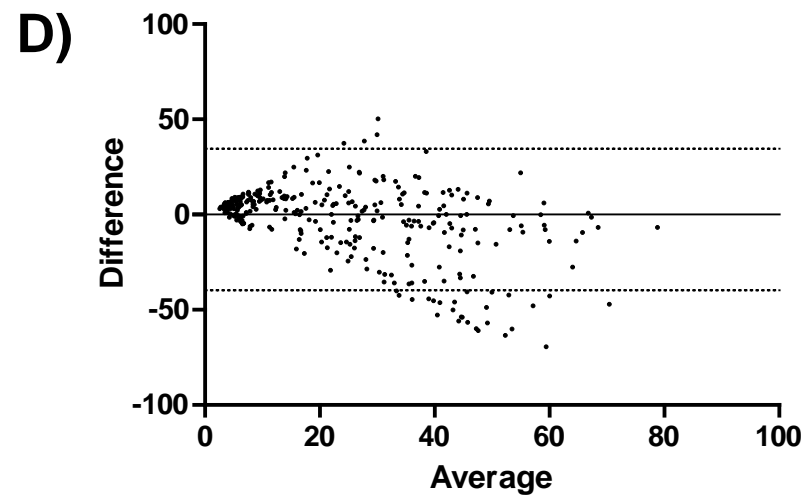

Supplement: Additional file 2 — Comparison of the concordance between pyrosequencing and the 450k array if the same CpG island is analyzed. For this purpose the mean methylation levels obtained by each method for a given CpG island were compared. Indicated are correlation coefficients according to Spearman (r) and linear regression coefficients (r2) [file 1756-0500-5-210-S2.pdf]

## cell lines

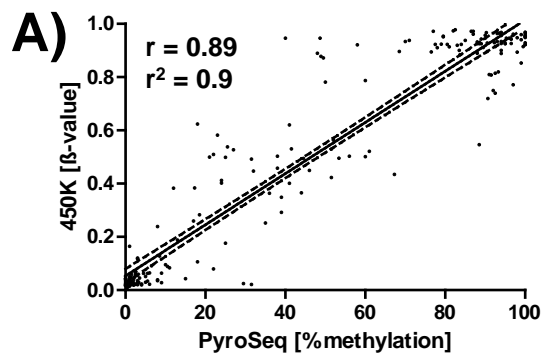

## primary tissue

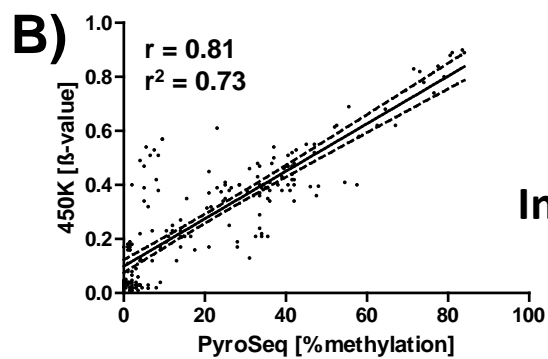

Infinium I

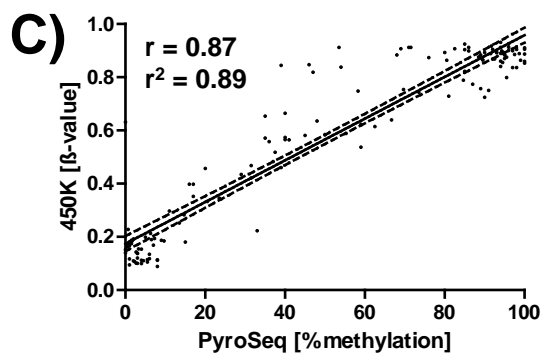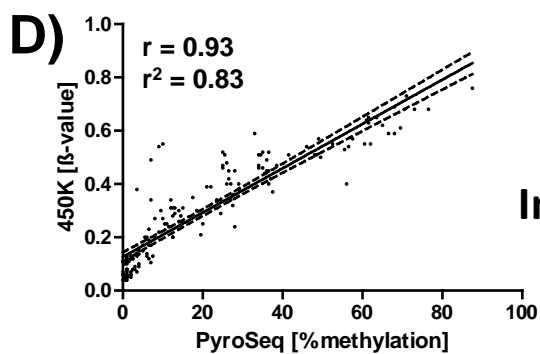

Infinium II

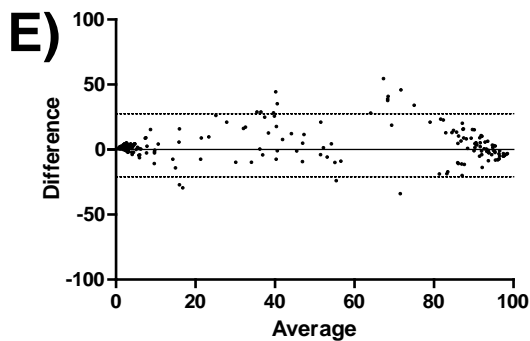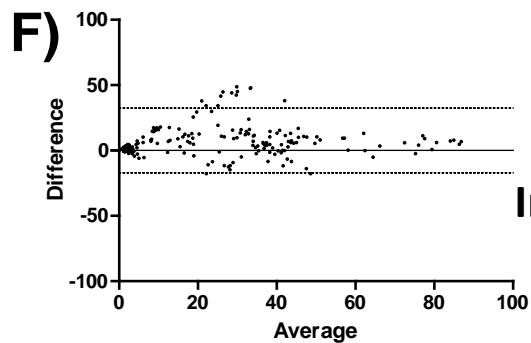

Infinium I

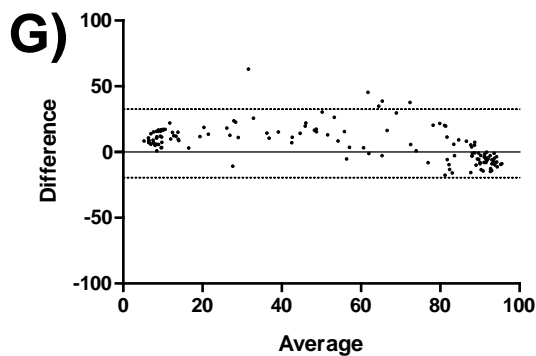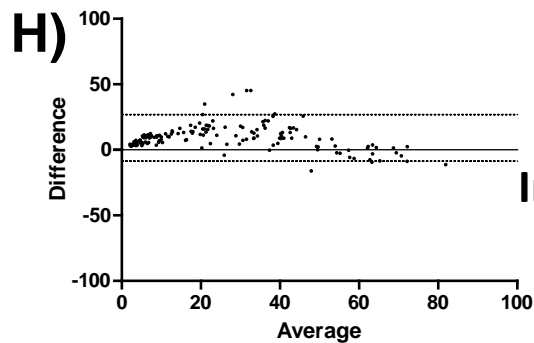

Infinium II

Supplement: 4 — Comparison of the concordance between pyrosequencing and the 450k array for Infinium I and II assays separately. Indicated are correlation coefficients according to Spearman (r) and linear regression coefficients (r2) [file 1756-0500-5-210-S4.pdf]

## Slide 1
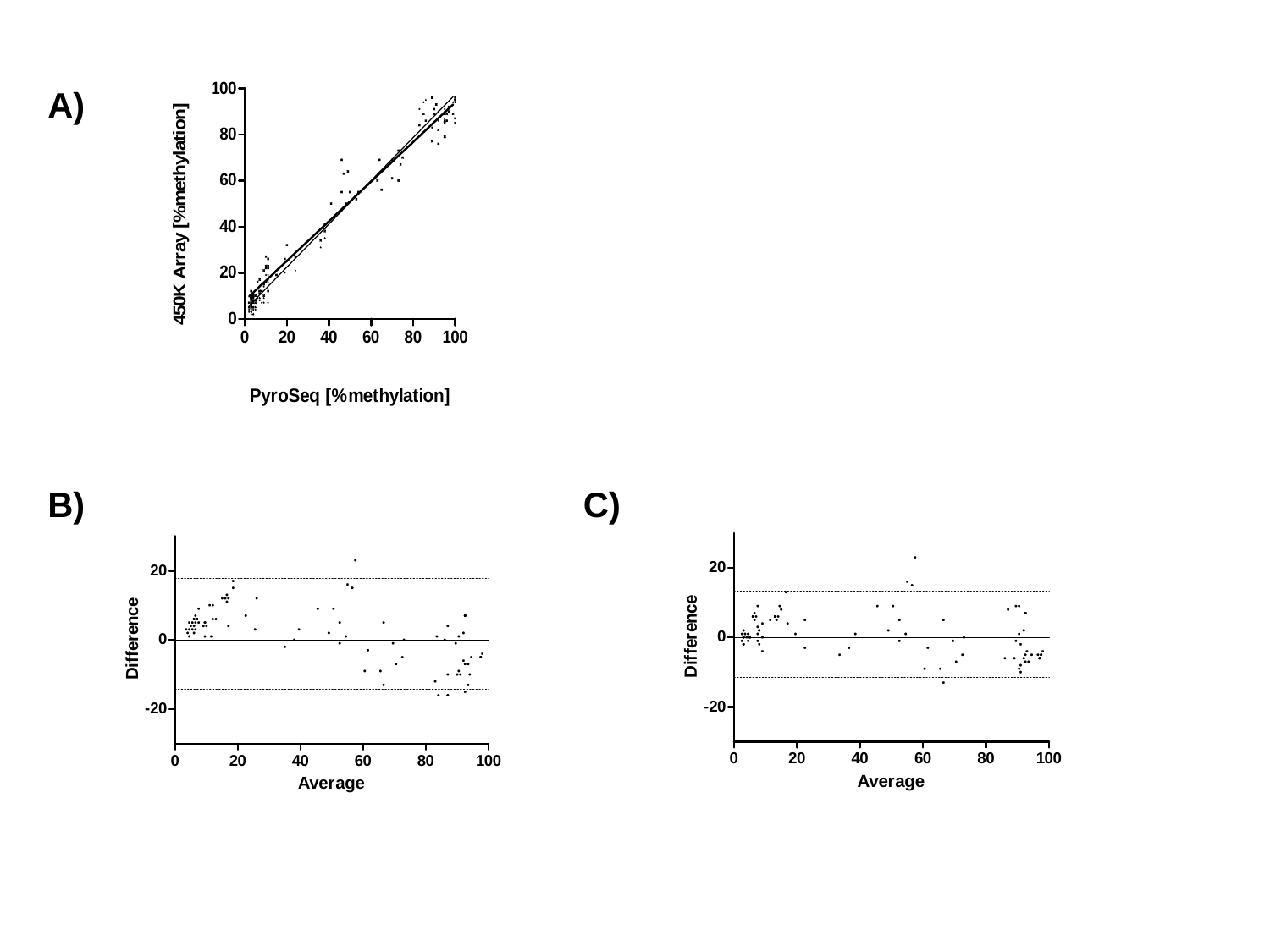

A)
B)
C)

Supplement: Additional file 5 — Scatter plot (A) and Bland-Altman-Plots without (B) and with (C) peak correction for the data from Dedeurwaerder et al. Table S2 [file 1756-0500-5-210-S5.ppt]
