# Supplementary material for: Quantitative cross-validation and content analysis of the 450k DNA methylation array from Illumina, Inc
Source: BMC Res Notes. 2012 Apr 30;5:210. doi: 10.1186/1756-0500-5-210 (PMC3420245; doi:10.1186/1756-0500-5-210)
Supplement: Additional file 3 — Coordinates for all individual CpG sites compared in Figure 1 [file 1756-0500-5-210-S3.pdf]

	Index	TargetID	MapInfo	Chr	Sample group	
1	1571	cg00066729	73633071	13	CL	
2	10610	cg00499838	61148684	20	CL	
3	11103	cg00522056	154335386	2	CL	
4	18782	cg00893636	37034840	3	CL	
5	22187	cg01052879	61809830	20	CL	
6	31944	cg01544807	42195539	8	CL	
7	36273	cg01773059	64761203	14	CL	
8	40498	cg01972979	133680446	X	CL	
9	44451	cg02169391	130126176	7	CL	
10	51172	cg02511809	112073544	5	CL	PT
11	68548	cg03387135	61809827	20	CL	
12	69196	cg03422070	2722082	11	CL	PT
13	76740	cg03801902	4381800	12	CL	PT
14	83513	cg04155289	94953770	7	CL	
15	94715	cg04743654	50378292	3	CL	PT
16	98226	cg04920951	67351271	11	CL	
17	101535	cg05096161	46114674	17	CL	
18	114187	cg05785947	68771059	16	CL	
19	140716	cg07194250	133680463	X	CL	
20	151357	cg07776419	133680476	X	CL	
21	154027	cg07921535	94285911	7		PT
22	157895	cg08133931	48636626	17	CL	PT
23	166926	cg08633479	57630524	6	CL	
24	186316	cg09750382	61148781	20	CL	
25	188528	cg09872616	130126182	7	CL	
26	189175	cg09910998	94285942	7		PT
27	189637	cg09938227	156390124	1	CL	
28	191737	cg10065153	101291602	14		PT
29	197481	cg10382221	46114672	17	CL	
30	200025	cg10528576	101192913	14	CL	PT
31	200977	cg10583931	75379800	5	CL	
32	202006	cg10639888	154335368	2	CL	
33	205123	cg10830518	111804752	6	CL	PT
34	205722	cg10866298	9760869	8	CL	
35	206893	cg10943497	101291571	14		PT
36	218741	cg11666921	2722062	11	CL	PT
37	228609	cg12234996	53254760	X	CL	
38	228713	cg12243267	57630536	19	CL	
39	238601	cg12877251	9760880	8	CL	
40	240171	cg12981137	131265575	10	CL	
41	243790	cg13218903	154335379	2	CL	
42	247514	cg13431205	48893174	13	CL	
43	250649	cg13575161	4381792	12	CL	PT
44	253230	cg13697378	68512845	1	CL	
45	257622	cg13917504	130131258	7	CL	
46	257694	cg13920367	68771035	16	CL	
47	261360	cg14092276	101192852	14	CL	PT
48	263641	cg14203179	57430663	20	CL	
49	273465	cg14687474	41278281	17	CL	

50	275055	cg14783814	98511792	1	CL	
51	279155	cg15028514	61809841	20	CL	
52	289005	cg15651941	2722084	11	CL	PT
53	294361	cg15980539	152128865	6	CL	
54	294588	cg15993083	4381788	12	CL	PT
55	294860	cg16006004	41278241	17	CL	
56	301895	cg16417926	53254711	X	CL	
57	302034	cg16424920	9760855	8	CL	
58	308756	cg16792632	64761208	14	CL	
59	309320	cg16829453	48636646	17	CL	PT
60	334053	cg18372208	41278263	17	CL	
61	334078	cg18373878	101192860	14	CL	PT
62	334954	cg18426655	14891118	X	CL	
63	336461	cg18513313	46970868	10	CL	
64	338247	cg18627360	61809834	20	CL	
65	345388	cg19086599	61148784	20	CL	
66	350927	cg19454999	41278357	17	CL	
67	354583	cg19678392	94953810	7	CL	
68	354840	cg19694923	68516374	1	CL	
69	357777	cg19856728	87963592	1	CL	
70	357868	cg19861138	154335384	2	CL	
71	358573	cg19903753	53254678	X	CL	
72	362602	cg20156391	61148764	20	CL	
73	363262	cg20206224	43543771	6	CL	PT
74	364850	cg20311501	112073502	5	CL	PT
75	381209	cg21384971	46114574	17	CL	
76	382702	cg21490561	37034825	3	CL	
77	383297	cg21526238	144329672	6	CL	
78	383438	cg21535000	9760877	8	CL	
79	387119	cg21778340	94285902	7		PT
80	395245	cg22333214	98511789	1	CL	
81	404836	cg22955387	133680468	X	CL	
82	416438	cg23658326	37034787	3	CL	PT
83	420805	cg23938220	112073538	5	CL	PT
84	422092	cg24007917	77272927	12	CL	PT
85	442774	cg25288140	41278341	17	CL	
86	443030	cg25306939	2722086	11	CL	PT
87	444743	cg25407198	130131268	7	CL	
88	445046	cg25425078	4381777	12	CL	PT
89	450411	cg25771013	25989735	7	CL	
90	454768	cg26064831	24711899	X	CL	
91	454920	cg26073933	156390102	1	CL	
92	455251	cg26094482	2722073	11	CL	PT
93	457999	cg26250609	67351273	11	CL	
94	464916	cg26649384	154334651	2	CL	
95	469522	cg26908876	2722076	11	CL	PT
96	473757	cg27150681	89618861	4	CL	
97	475850	cg27282761	74806951	5	CL	PT
98	477839	cg27393730	94285951	7		PT
99	479992	cg27526317	53254742	X	CL	

Sample group: CL - Cell lines PT - Primary Tissue
