# Supplementary material for: Quantitative cross-validation and content analysis of the 450k DNA methylation array from Illumina, Inc
Source: BMC Res Notes. 2012 Apr 30;5:210. doi: 10.1186/1756-0500-5-210 (PMC3420245; doi:10.1186/1756-0500-5-210)
Supplement: Additional file 6 — Primary human specimens used in this study [file 1756-0500-5-210-S6.pdf]

Supplementary Table S1 Patient material

ID	age (yr)	Gender	Tissue
1	44	female	invasive breast cancer
2	92	female	invasive breast cancer
3	62	female	invasive breast cancer
4	60	female	invasive breast cancer
5	57	female	invasive breast cancer
6	55	female	invasive breast cancer
7	90	female	invasive breast cancer
8	68	female	invasive breast cancer
9	50	female	invasive breast cancer
10	64	female	invasive breast cancer
11	33	female	invasive breast cancer
12	32	female	invasive breast cancer
13	48	female	invasive breast cancer
14	37	female	invasive breast cancer
15	52	female	invasive breast cancer
16	25	female	invasive breast cancer
17	50	female	invasive breast cancer
18	32	female	invasive breast cancer
19	24	female	normal breast tissue
20	28	female	normal breast tissue
21	47	female	normal breast tissue
22	32	female	normal breast tissue
