# Supplementary material for: Quantitative cross-validation and content analysis of the 450k DNA methylation array from Illumina, Inc
Source: BMC Res Notes. 2012 Apr 30;5:210. doi: 10.1186/1756-0500-5-210 (PMC3420245; doi:10.1186/1756-0500-5-210)
Supplement: Additional file 7 — Sequence of all primers used in this study [file 1756-0500-5-210-S7.pdf]

Gene	Forward primer	Reverse Primer	Sequencing Primer
APC	GGA GAG AGA AGT AGT TGT GTA ATT T	ACT ACA CCA ATA CAA CCA CAT ATC	TTA GGG TGT TTT TTA TTT T
BRCA1	TTG TGG GGT GAA TTT AAT ATG G	CCC CAA AAA TCT AAA ACA AAT AAT CTT ATA	GGA TAA AGA TAG TAA TTA GT
			GAT TAT TTT ATT GAT TGG TG
CCND2	GTA TTT TTT GTA AAG ATA GTT TTG ATT	CCA AAC TTT CTC CCT AAA AAC	ATA GTT TTG ATT TAA GGA TG
CDH1	AGA TTT TAG TAA TTT TAG GTT AGA GG	CTA ATT AAC TAA AAA TTC ACC TAC C	ATT TTA GGT TAG AGG GTT AT
CSRP2	GTT TTT TGT TTT TTG GTG GTT AG	CAA AAA ATC CRA AAT CCC AAA C	AGY GGG TAG AGG GTA G
DAPK1	TTT YGG AGT GTG AGG AGG ATA	AAA ACR CAA ATC CCT CCC AAA	AGT GTG AGG AGG ATA GT
DIRAS3	TGG ATT AGT TTT TAG ATT GTT GTA GAT GT	CCC CAA AAA CTC ACT CCT CC	GGT TAG TTT TTT ATA GTT GGT
DLKp	TTT GTG TTT TTT TGG GTA TTT AAT TAT TG	TTA ATA CAC CTT CCC TCA CAC TAT ACA ACA	GTT TTT TTG GGT ATT TAA TTA TTG T
ER1	TTY GTT TTG GGA TTG TAT TTG T	ACC TAA AAA AAA AAC ACA ACC C	GGG ATT GTA TTT GTT TT
ER2	TTT TTG TGG GTG GAT TAG GAG T	CCC CTT CTT CCT TTT AAA AAC	GTG TTT TTA TTT AGA GGT TA
FANCB	TTT TTG GTT GGT TTT TAG GTT	AAC CCC ATC CTA CCT CAA C	GGT TGG TTT TTA GGT TT
GNASXL	AGT GGG AGG AGG GGG TTT AGT TAA AG	AAC CCC ACC AAC CTA ACC AAA AAA AT	TTT TTA GGG AGA AAA GTG
GSTp1	GGG GAG GGA TTA TTT TTA TAA	AAT TAA CCC CAT ACT AAA AAC TCT	GGA TTA TTT TTA TAA GGT
Hin1	TGT TTG TTT TTA GAG GGT TTT AG	TAA AAA AAC CCT ACC CRC TCC	GGT AGG GAA GGG GGT
hsa-mir-1-1	TGG GGT TAA ATT TAT TTT GAA TTT G	ACT CCC CAA CAA AAA CCT ACA C	GGG GGT AGG GAT TTT
hsa-mir-124A-1	TTT TTT TAG GAG AAA GGT TTT	AAA ACT CCA AAC CCC TC	GAA TGG GGT TGG TTG AGT AT
hsa-mir-124A-3	AAA GGG GAG AAG TGT G	CAT ACC TTA ATT ATA TAA ACA TTA AAT C	AAA CCC TCA AAA CTA AAA C
hsa-mir-137	AGT TTA GTT TAT TTT TAG GTA GGG G	AAA ATC AAA AAA CCA AAC TAC C	GAG TAG TAA GAG TTT TGG TG
hsa-mir-148A	TGG GTA TTT GTT TTT GTT GAT T	ACT ACA CTT AAA CCC CCT CTA ACC	CAA CTA AAA CAA AAA AAA ATA ACT C
hsa-mir-152	GGA ATT TTG TGT TAT TTT TGA TTG	CCA AAC AAA TAT TCC ACT ACA AAC	TTC TTC CAA CTA ATC CCT AAC
hsa-mir-424	GGT AGT ATT TTT GGT TAG GTT GG	AAA CAA TAC CCC AAA ACA CC	TTT GGT TAG GTT GGG
hsa-mir-503	GTA GTA TTT TTG GTT AGG TTG GG	AAA CAA TAC CCC AAA ACA CC	TCA CTA CAA AAC TAT TCC C
hsa-mir-9-1	TTG TAT TTT TTG GTG TTG GTT AG	CTC RTA TCC CTT CCC TCC TAC	AAA TAA CCC CAT ACA CTA C
hsa-mir-9-2	GGA AGA GAT GTT GAT TGA GAA AA	TAA TCA ACC AAC TAC CCC AC	GGG ATT GTT GTA ATG TTG
KLF11	TTG TTT TYG TTT TTT GGA TGG AG	AAA AAC AAA AAC CAT ACT ATT TAT TTT C	ATT YGT ATT ATA GTT TAG TAA AGG
KLF5	TGT TTT TTT TTT TGT TTA TAG GTT GG	CCT CTA TTT CAC CCA ACT CC	GGT YGT TTA GGT TTG GT
KvDMR	AGG GAA GTT TTA GGG TGT GAA TTT TTA GAG	CCA AAC CAC CCA CCT AAC AAA AAA C	TGG TAA TGT TTG GTA TTT
MafB	TTT AAT TTA ATT TTG TGG GGT GGT	CCC CCA ACT ACT AAC AAA AAC TAC	AAA TTA AAT GTT GGA AGA G
MAL	GTA TAG TTG GGA GTA ATT TTT TA	CCA CAA ACT AAA AAT CTA CAA TA	TAG TAT TAA GTA GAG AGG T
MEG3	GTT AGG TAG GGT GAA TTT AGG TAT AAT GTG TG	AAC AAC CTC TAA CTT ACA TCT AAA AAC CAA TT	ATG GTT AAG GTG GGG TTG
	GTA TTT TGA TTT TTG YGA GAG GAT	ATC CCC ACA CAC ATA CCC TTT	CTT TAA AAA AAC CCC AA
MEST	AAG GGG GTT TTG TTT TTT TAA TTG TG	AAC CCC ACA AAC TAC CCA CAA ACC	TTG TTG TAA AGG AAA TTT
	TTT TTG GGA ATA GGG TGA AGG T	AAC CAC ACC CCC AAA AAA AAC TAA T	AGA ATT TTT GGT TTT AGG
MGMT	GTT TYG GAT ATG TTG GGA TAG	AAC CAC TCR AAA CTA CCA CC	TGG TGA GTG TTT GGG T
Mint1	TTY GGG TTG GGT ATT TGG ATT T	TTC AAA CTC TCT CAA CAC TTA C	ATA GTT GTT TTA GGT TTA ATT
MINT12	GGT TTT AGT TTT GAG GAT TAG G	TCA CTA ACC TTT AAA TAA TCT AC	AGG GTT TAG AAG ATT TTA G
Gene	Forward primer	Reverse Primer	Sequencing Primer

Mint17	TGA AAG GGG TTA GGT TGA GG	TTC TAC CTC TTC CCA AAT TCC	AGG AAG AAT ATT TTG GAT G
Mint27	GGT TTG AGG AGG ATT TGG GA	AAA AAC AAA ACT ACA ACC TTC AC	TTT TGG TTA TTA GGT GTG A
Mint31	GTT TAG GGG TGA TGG TTT TAG	AAA CAC TTC CCC AAC ATC TAC	GTG GTG ATG GAG GTT AT
MKRN3	AAT TAT AGG TAA GGA AAG GGA GGA TGT AGT	AAA CCT ACC CCA ACC TCA AAA AAA A	ATT TGT AGA GGT TTT AGG
MLH1	TTT TAA AAA YGA ATT AAT AGG AAG AG	AAA TAC CAA TCA AAT TTC TCA ACT C	AAA YGA ATT AAT AGG AAG AG
			AAG YGT ATA TTT TTT TAG GTA G
NAP1L5	AGG GTA GTA ATA GGA GGA ATT TGG TGT AGT	AAA CTC CTC AAC CAT CTA ACC AAC C	GAT AGT GGG AAG TTA GTT AAG
NTRK2	TAG AGA GGG AGA GTG TTT TAA	AGG AAT TTG RGT TTT AGA GAG TT	TGG TGT TGG GGG TTT TA
OPCML	TAG GTA TTT TTA GTT YGG TGG	CCA CTT TCT CCC RAT ACC	GGA GGT TAA GGT GGG
p15	GTT GGT TTT TTA TTT TGT TAG AG	AAC TCA ACT TCA TTA CCC TCC	GGG GCT AGT GAG GAT TT
p16	GAG GGG TTG GTT GGT TAT TAG A	TAC AAA CCC TCT ACC CAC CTA AAT	TGG TTA TTA GAG GGT G
Peg10	TTG GTT TAG GTG TGG GAT TTT T	AAA CAT TCT AAA ATA CTA CTC CAT CTC	TCC ATC TCC CRC AAC TCC
POL A	TTT TTA AGG GGT TAT TAT AGG G	CAA CCA ACC CAA AAC TAA C	AGG GTT ATT ATA GGG
POL B	TTT TGA TAT TGG TTG GTA GGT G	ATC AAA ACT TTC TCT AAA AAT ACC	TGT GYG GGA GGA TTT TTG
POLH	AGA TTA TTY GTT GGT ATY GGG	CCT AAA CTC AAC CTA CAC AC	GAA GTT GGA GGA GGA G
POLK	TTG TAG GTG AGT TTG GTT AGG	TAA AAA AAC RAA AAC CRA TCT CC	TTG TTT ATT TTA TTG AGG ATT
POLZ	TTT TTT GTT GGA TGT GAA ATG G	CTC ATA AAT AAA CTC CRA AAA AC	GAG GTA AGT GTG AGT AT
PON1	ATA GTT TGG ATT TAA TTT TTT GGG GG	CAA ACA AAC AAA ACC TCC TAA CCC	TTA TTT TTA AGA GGG TGA
Rassf1A	AGT TTG GAT TTT GGG GGA GG	CAA CTC AAT AAA CTC AAA CTC CCC	GGG TTY GTT TTG TGG TTT
RB1	GGT AGG GTA GTT TTG GAA ATG TTT AAG	AAC CAC AAA CCC TTA CCC	AGT TTT GGA AAT GTT TAA GAT
Rev1	GTT AGT GTT TGT ATT TGT GTT TTT	CCC TAT TAT TCT ACA ACC YCC	TTT AAG GYG GGG TAG G
RPRM	AAG AAG TAA GTA ATA AAA AGT AAG ATT AGG T	ACT TAA CRA AAC TAA ACC AAC C	TTT TTT TAT TTT TTA AGA GTA G
	TGT GTT TTT TAT TAT TAG AGA TTG GG	ACT ACC AAA CAT AAA AAC TTT CAA AA	CAT AAA AAC TTT CAA AAA AC
Sall3	TTT TAG AGT AGT TTG YGT TGG G	AAA ACA AAA TAC RAA CTC TAC CC	GTA AGT TTT TTT AGA TTT TTG
SFRP1	TTG GGG ATT GYG TTT TTT GTT	ACT CTA CRC CCT ATT CTC C	GAG GTT TTT GGA AGT TTG
SLC22A3	GTT GTT TTT TGG GGA AAG TTG GGT	TCC TTT TAA TCC CTA CCA ACA ACC TA	GTA AGG GTT AAG GGT TGG A
SMCX	AGA AGG GAT AGA GAA TAT TGG T	ACT AAA AAA AAA TAA TTC ATT ACA AC	GGA TAG AGA ATA TTG GTG
SOCS1	GTG AAG ATG GTT TYG GGA TTT	CAA CRA AAC CCC CAA CAT AC	GTT TTT ATT TGG ATG GTA G
USP29	GAG ATG GAA GTT GGT AGG ACG TAG	GCG AAA CCT CAC GAA TAA CG	TGA TTG TAT TTG GGT TT
ZAC	GGT TGA ATG ATA AAT GGT AGA TGT	ACC TTA ACT TTA CCC CCA C	TGG TAG GAG GAG GTT T
